# Precisely tuning the longitudinal localized surface plasmon resonance of gold nanorods *via* additive-regulated overgrowth

**DOI:** 10.1039/d0ra00579g

**Published:** 2020-03-27

**Authors:** Suyan Wang, Qinlu Lin, Weizhen Xu, Qingxiao An, Rongju Zhou, Cheng-Ju Yu, Dong Xu, Zhiqin Yuan

**Affiliations:** National Engineering Laboratory for Rice and By-products Further Processing, Central South University of Forestry &Technology Changsha 410004 China philip198349@gmail.com +86-731-8562-3240; Department of Applied Physics and Chemistry, University of Taipei Taipei 10048 Republic of China; State Key Laboratory of Chemical Resource Engineering, College of Chemistry, Beijing University of Chemical Technology Beijing 100029 China yuanzq@mail.buct.edu.cn +86-10-64411957

## Abstract

Gold nanorods (GNRs) with desired longitudinal localized surface plasmon resonance (LLSPR) and strong scattering intensity are important for extending their practical applications in bioimaging and sensing. Herein, a simple additive (HCl and Na_2_S)-regulated overgrowth approach has been proposed for preparing GNRs with tunable LLSPR. In this approach, HCl is used to slow down the growth reaction rate by changing chemical equilibrium, while Na_2_S is utilized to halt the reaction when LLSPR is reaching the expected wavelength under monitoring by a UV-Vis spectrometer. Under optimal conditions, GNRs with an LLSPR range from 850 to 650 nm could be facilely prepared with a high precision of 3 nm deviation. The TEM images reveal that GNRs have high monodispersity, displaying an increase in both length and diameter but a decrease in the aspect ratio. With the increase in size, the produced GNRs show enhanced scattering intensity and are applicable for single nanoparticle imaging due to the enlarged absorption and scattering cross-section and improved matching efficiency toward the CCD response.

## Abstract 2

An additive-regulated overgrowth strategy for preparing gold nanorods with precise longitudinal localized surface plasmon resonance is proposed.

## Graphical abstract

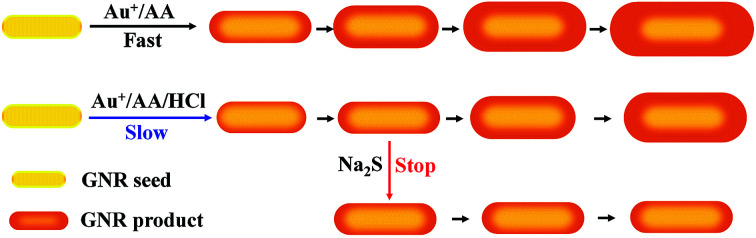

## 1. Introduction

Gold nanorods (GNRs) with unique optical and surface properties have attracted increasing interests in sensing, phototherapy, gene and/or drug delivery, and bioimaging.^1–6^ As the most intriguing and important feature of GNRs, the longitudinal localized surface plasmon resonance (LLSPR) originating from the collective oscillations of free electrons and confined to the nanoparticle surface can range from visible to near-infrared regions.^7^ Unlike the LSPR of spherical gold nanoparticles, the LLSPR of GNRs is only dependent on the aspect ratio (AR, length/diameter) and is important for their further applications.^8,9^ For example, GNRs with LLSPR in the range of 750–1300 nm are more favorable as *in vivo* imaging probes because of the low background from tissues in this wavelength window.^10,11^ Recently, the anisotropic characteristics have made GNRs promising optical tools for understanding the biological events at the single particle level through monitoring both translational and rotational motions.^12,13^ Wavelength-dependent photo collection efficiency of optical detectors (*e.g.*, charge-coupled devices (CCDs) and photomultipliers) and a high signal-to-noise ratio can be realized when LLSPR matches the detector's optical response curve.^14^ Besides, for photothermal therapeutic applications, shorter or longer LLSPR than the laser wavelength would reduce the light-heat efficiency and decrease the therapeutic effect.^15^ Therefore, the development of facile routes to repeatedly synthesize LLSPR-tunable GNRs is of significance to construct versatile GNR-based optical platforms.

Toward this goal, much efforts have been dedicated to the alteration of AR and geometry with various synthetic strategies. In general, a seed-mediated growth method, with the advantages of simplicity, high yield, size tunability, and monodispersity, has been widely applied in the preparation of GNRs with designable LLSPR properties.^1,16^ For example, GNRs with a supreme LLSPR of 981 nm and uniform morphology are synthesized under alkaline conditions by replacing ascorbic acid (a reducing agent) with H_2_O_2_.^17^ With the addition of aromatic additives during seed-mediated synthesis, GNRs with tunable LLSPR beyond 1000 nm and improved purity are obtained.^18,19^ However, seed-mediated synthesis sometimes shows low reproducibility, limiting their applications.^20^ To overcome this concern, post-synthesis, that is, subjecting the pre-synthesized GNRs to further oxidation or overgrowth has been applied in the production of LLSPR designable GNRs.^21–25^ For instance, the length of GNRs can increase/remain with/without adding thiol molecules.^26,27^ In comparison to oxidation, the overgrowth strategy is more valuable to yield GNRs with high light absorption and scattering efficiency by increasing their sizes.^26^ Despite the tunable LLSPR of GNRs with the overgrowth strategy, tuning precision is still a problem. Thus, the exploration of a simple approach for precisely and reproducibly tuning the LLSPR of GNRs is still appealing.

**Scheme 1:**
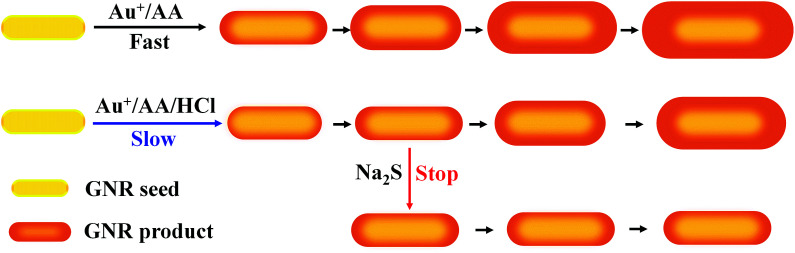
Tuning LLSPR of GNRs through decreasing and halting the overgrowth with HCl and Na_2_S.

In this study, a simple and reproducible strategy for the preparation of GNRs with the precise tuning of LLSPR through HCl and Na_2_S-regulated overgrowth was proposed. The addition of HCl can slow the overgrowth reaction rate by altering chemical equilibrium, while the addition of Na_2_S leads to the depletion of the gold element due to the formation of Au_2_S in the solution, which terminates the overgrowth process, as shown in Scheme 1. By simply changing the amount of HCl and Na_2_S, GNRs with tunable LLSPR from 650 to 850 nm could be easily produced with a precision of 3 nm. Single nanoparticle imaging was also conducted with darkfield microscopy, proving that GNRs with LLSPR at 650 nm showed the strongest scattering because of the largest cross-section and the best matching of LLSPR with the CCD response. This work provides a promising strategy to produce desired GNRs with precise LLSPR and strong scattering intensity, which may facilitate the assays at the single-nanoparticle level.

## 2. Experimental

### 2.1 Chemicals

Hydrogen tetrachloroaurate (iii) trihydrate (HAuCl_4_·3H_2_O), cetyltrimethylammonium bromide (CTAB), sodium sulfide nonahydrate (Na_2_S·9H_2_O), sodium hydroxide (NaOH), and hydrochloric acid (HCl, 36–38%) were purchased from Sinopharm Chemical (Shanghai, China). Sodium borohydride (99%), silver nitrate (AgNO_3_), l-ascorbic acid (C_6_H_8_O_6_, AA) were obtained from Sigma-Aldrich. All the chemicals were of analytical grade, and the solutions were prepared with deionized water (18.2 MΩ cm).

### 2.2 Characterizations

UV-Vis absorption spectroscopy was performed on a UV-1800 Spectrometer (Hitachi, Japan). TEM images were obtained using a JEM 1230 transmission electron microscope operating at 100 kV (JEOL, Japan). Single GNR images were acquired with an NI-U upright microscope (Nikon, Japan), which was equipped with a 100 W halogen tungsten lamp, an oil immersion darkfield condenser (NA 1.20–1.43), a 40× plan fluor objective, and a DP 73 camera (Olympus, Japan).

### 2.3 Synthesis of GNR seeds

GNR seeds for further overgrowth were prepared through a seed-mediated method according to previously reported methods.^28,29^ Briefly, small spherical gold nanoparticles with a diameter of around 3 nm were initially obtained by reducing Au^3+^ with a strong reducing agent. Then, 48 μL of ice-cold 0.010 M NaBH_4_ was injected into 8 mL solution containing 0.1 M CTAB and 0.00025 M HAuCl_4_ under vigorous stirring. Then, this solution was kept at 28 °C for at least 2 hours prior to use. For GNR-seed synthesis, 412 μL of 24.28 mM HAuCl_4_, 0.5 mL of 4 mM AgNO_3_, and 105 μL of 0.1 M AA were added to 20 mL 0.1 M CTAB solution in order. After shaking this solution to make the colour change from bright yellow to colourless, 60 μl of the nanoparticle solution was further injected. The mixture was shaken for 20–30 seconds again and then kept at 28 °C for 4 h. In order to assess the reproducibility of GNR synthesis, three researchers repeated the experiment with the same recipe.

### 2.4 HCl and Na_2_S on the overgrowth of GNRs

Understanding and controlling the dynamics of GNR overgrowth in solution are essential to attune the LLSPR of GNRs. The effect of pH on the dynamics was examined by adding HCl or NaOH. First, 72 μL of 24.28 mM HAuCl_4_ and 26.7 μL of 0.1 M AA were added to 3 mL 0.1 M CTAB solution in a 5 mL cuvette, followed by shaking until the solution became colorless. Then, 0 μL, 30 μL, and 300 μL aliquots of 1 M HCl or 30 μL of 1 M NaOH was injected to adjust pH, and extra distilled water was added to make up the final volume to 3.3 mL. After adding 375 μL GNR-seed solution, the UV-Vis spectra of the mixtures were monitored using a UV-Vis spectrometer at an interval of 30 seconds. To obtain the optimum amount of Na_2_S to halt the GNR growth when LLSPR reaches 800 nm, 17.5 μL, 35 μL, 70 μL, 140 μL, 280 μL, and 560 μL of 0.1 M Na_2_S solutions were injected. The UV-Vis spectra of GNR solutions before and after the addition of Na_2_S at 1.0 min and 15 min were measured. Finally, GNRs with LLSPR of 650 nm, 700 nm, 750 nm, 800 nm, and 850 nm were prepared by adding 30 μL of HCl to slow the growth and 140 μL of 0.1 M Na_2_S to stop it.

### 2.5 Single GNR imaging

To perform single-particle imaging, 10 μL of 1000-fold ultrapure water-diluted GNR sample was dropped on a clean slide. After putting a clean cover glass on it, the sample was immediately examined under a microscope and images were acquired with the exposure times of 10 ms, 50 ms, 100 ms, and 500 ms.

## 3. Results and discussion

### 3.1 HCl and Na_2_S-regulated overgrowth of GNRs

**Fig. 1:**
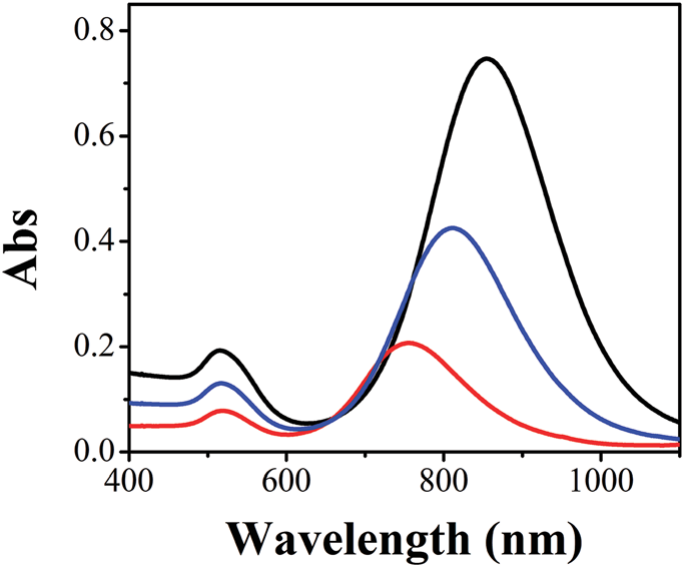
UV-Vis spectra of GNRs prepared by three researchers using the same formulation: 4.8 × 10^−4^ M HAuCl_4_, 9.5 × 10^−5^ M AgNO_3_, 5.0 × 10^−4^ M AA in 20 mL 0.1 M CTAB solution and 60 μL of spherical small nanoparticles.

The problem of traditional seed-mediated GNR synthesis is variable LLSPR even with the same protocol.^20,30^ At the starting point, GNRs using 4.8 × 10^−4^ M HAuCl_4_, 9.5 × 10^−5^ M AgNO_3_, and 5.0 × 10^−4^ M AA in 20 mL 0.1 M CTAB solution and 60 μL of small gold sphere nanoparticle solution were prepared by three researchers. The UV-Vis absorption spectra of the resulting GNR solution were measured (Fig. 1). Obviously, among these GNRs, the largest wavelength of LLSPR was 854 nm, while the smallest one was 760 nm. The deviation achieved was nearly 94 nm. The absorbances were also apparently different, where the highest absorption intensity of the GNR solution was three times the lowest value. Similar results were also reported previously.^20^ The reproducibility of the shape and morphology of GNRs still remains an issue since these are highly dependent on the reaction conditions and the residues of some organics may cause such differences.^20^ Another important reason is that NaBH_4_ can react with water to produce H_2_ and form bubbles, which affect the final amount of NaBH_4_ used for preparing spherical gold nanoparticles. Therefore, the LLSPR of the as-prepared GNRs is inevitably variable and cannot be anticipated; correspondingly, it is difficult to synthesize GNRs with particular LLSPR using controlling recipes.

**Fig. 2:**
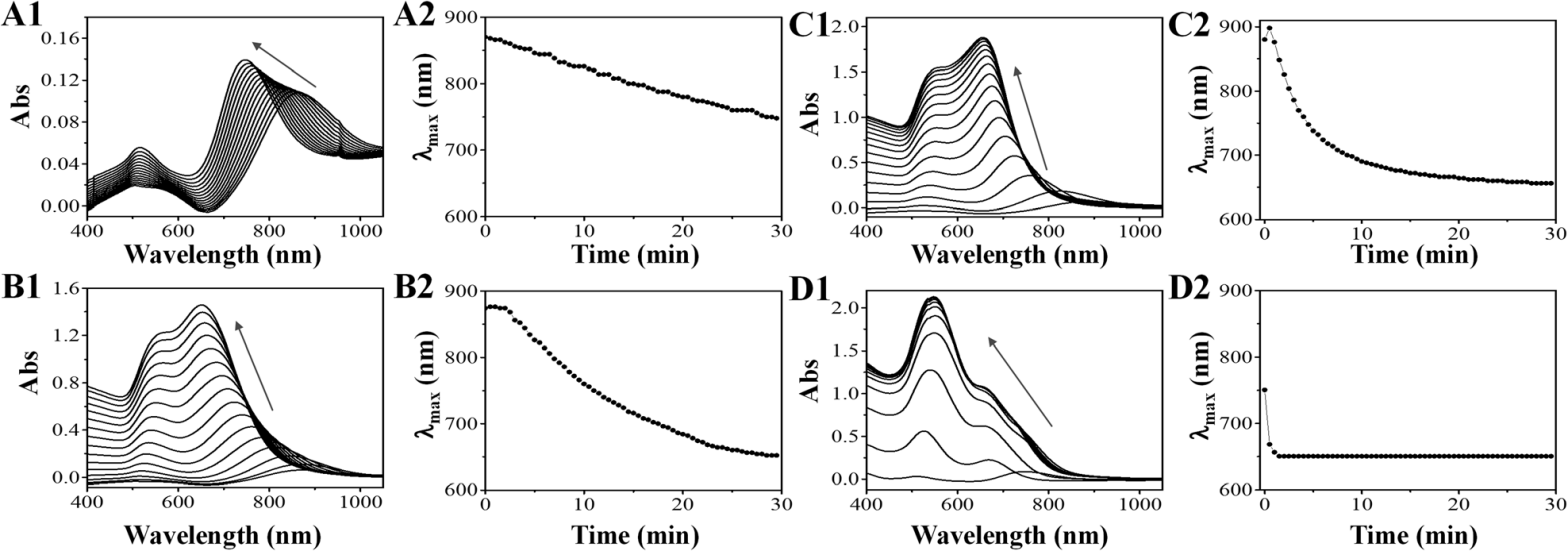
The UV-Vis spectra of GNRs (1) and the LLSPR position (2) *versus* time in the presence of 300 μL (A), 30 μL (B), 0 μL (C) of 1 M HCl and 30 μL of 1 M NaOH (D), respectively.

Since the overgrowth of GNRs results in continual blueshifts in LLSPR,^26^ we herein proposed an extensively applicable routine to repeatedly prepare GNRs with specific LLSPR, that is, halting the GNR overgrowth at a particular LLSPR, and this was monitored with a UV-Vis spectrometer. Controlling the kinetic dynamics of the GNR overgrowth process is promising to obtain GNRs with desired LLSPR. The chemical reaction of GNR overgrowth can be briefly described as follows:
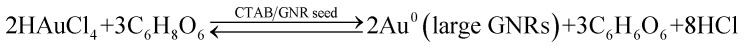


The reaction rate is dependent on the concentration of both reactants and products. Based on this chemical reaction, the forward reaction rate is simply described as *k*[HAuCl_4_]^2^[C_6_H_8_O_6_]^3^ and the backward reaction rate is *k*_−1_[C_6_H_6_O_6_]^3^[HCl],^8^ where *k* and *k*_−1_ are the rate constants (Au^0^ (large GNRs) is considered as a solid). At equilibrium, the forward and backward rates are equal; thus, we can obtain a rough equation, *i.e.*, *k*[HAuCl_4_]^2^[C_6_H_8_O_6_]^3^ = *k*_−1_[C_6_H_6_O_6_]^3^[HCl]^8^ and the equilibrium constant is expressed as follows: *K* = *k*/*k*_−1_ = [C_6_H_6_O_6_]^3^[HCl]^8^/[HAuCl_4_]^2^[C_6_H_8_O_6_]^3^. However, after the introduction of HCl at the beginning, the backward rate was higher initially, causing the production of Au^0^ to decline and the overgrowth of GNRs to slow down. The final equilibrium state would be altered, inducing decrease in the reduction in Au^0^. The UV-Vis spectra of GNRs during the overgrowth in 30 minutes with the addition of different amounts of HCl and NaOH were measured immediately after the addition of GNR seeds, as shown in Fig. 2. After the overgrowth, the LLSPR of GNRs blueshifted and the absorbance of their solutions increased, but the dynamic processes were slightly different. Without HCl or NaOH (pH = 3.05), the LLSPR would initially redshift by 24 nm in the first one min and then gradually blueshift to around 650 nm (Fig. 2C). Probably, the fast deposition of Au onto the end of GNRs leads to a small increase in AR at the start.^31^ With the addition of 30 μL of 1 M HCl (pH = 2.72), the LLSPR remained unchanged in the first 2 min, but the absorbance continually went up (Fig. 2B). Then, the LLSPR blueshifted slowly to 650 nm. Interestingly, with the addition of 300 μL of 1 M HCl (pH = 1.98), the LLSPR blueshift went on linearly but more slowly during the overgrowth (Fig. 2A). The average blueshift rates for the LLSPR of GNR solutions with the addition of 300 μL, 30 μL, and 0 μL of 1 M HCl in the first 5 min were 4.8 nm min^−1^, 14.2 nm min^−1^ and 40.6 nm min^−1^, respectively. In contrast, with the addition of 30 μL of 1 M NaOH (pH = 9.65), the overgrowth was completed within 2 min. The absorbance around 520 nm was higher than LLSPR, indicating the formation of a large amount of spherical gold nanoparticles because of the strong reducing ability of AA under basic conditions. The slow changes in LLSPR suggest the possibility of precisely tuning GNRs with the designed AR. The rate of 40.6 nm min^−1^ (without the addition of HCl) was too fast to stop the LLSPR of GNRs at a precise wavelength. However, too much HCl (300 μL, 1 M) only triggered a slight increase in absorbance, indicating a minor increase in the GNR size although the shift in LLSPR was slow. As a consequence, 30 μL of 1 M HCl (8.16 mM) was optimal to reduce the growth of GNRs and beneficial for producing GNRs with specific LLSPR.

**Fig. 3:**
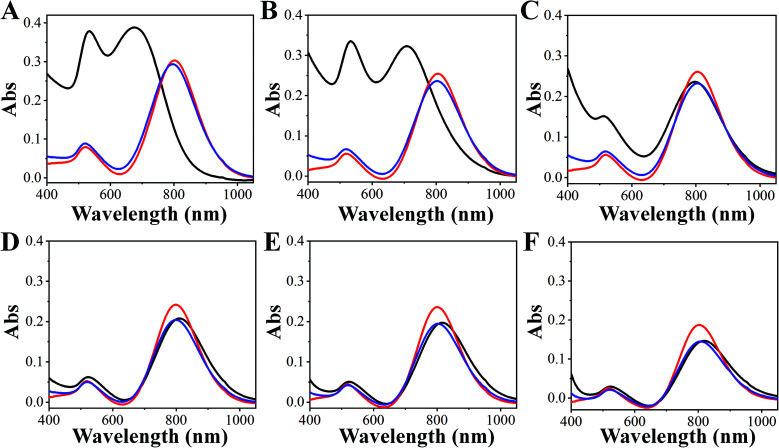
The effect of sulfide ions on the overgrowth of GNRs. The UV-Vis spectra of GNR solutions before (red line) and after the addition of sulfide ions at 1.0 min (blue line) and 15 min (black line) were obtained. (A)–(F) correspond to 17.5 μL, 35 μL, 70 μL, 140 μL, 280 μL, and 560 μL of 0.1 M Na_2_S.

Sulfide ions (S^2−^) have strong affinity for metal ions and can react with gold ions to form Au_2_S and Au_2_S_3_.^32–34^ Thus, the introduction of S^2−^ depleted gold ions and stopped the overgrowth of GNRs immediately. To understand the effect of S^2−^ on the overgrowth of GNRs, the UV-Vis spectra of GNR solutions with the addition of different amounts of Na_2_S when LLSPR reached 800 nm were monitored at 1.0 min and 15 min windows. As shown in Fig. 3A and B, the addition of 17.5 and 35 μL of 0.1 M Na_2_S is insufficient to stop the growth, and the LLSPR of GNRs still undergoes blueshifts to 676 nm and 708 nm, respectively. A possible reason was that gold ions were not totally exhausted by S^2−^. We also noticed that the absorption intensity of transverse surface plasmon resonance (around 520 nm) underwent a huge increase after 15 min of reaction. The main reason is that with the insufficient addition of S^2−^ ions, the newly formed Au_2_S and Au_2_S_3_ act as the nuclei for further gold deposition and generate spherical gold nanoparticles. Increasing the S^2−^ concentration would regress such further overgrowth. When 70 μL of Na_2_S was added, the growth was completely terminated and the LLSPR of GNR remained steady (Fig. 3C). Surprisingly, further increasing the S^2−^ concentration could result in a redshift in LLSPR. For example, after using 140 μL, 280 μL, and 560 μL of 0.1 M Na_2_S, redshifts of 6 nm, 14 nm, and 16 nm, respectively, were observed. Such unusual redshifts are attributed to the formed Au_2_S_3_ layer on the surface of GNRs with extra S^2−^ ions.^33,35^ The formed Au_2_S_3_ coating layer changed the refractive index of GNRs and led to a redshift in LLSPR. Interestingly, the S^2−^-terminated GNRs showed high stability. After centrifugation and redispersion, the LLSPR of these GNRs had no shift during a 2 month storage period, demonstrating their high stability.^33^ Thus, 70 μL of 0.1 M Na_2_S (a final concentration of 1.83 mM) was adopted to stop GNR growth and obtain LSPR at any wavelength.

### 3.2 Synthesis GNRs with precise LLSPR

**Fig. 4:**
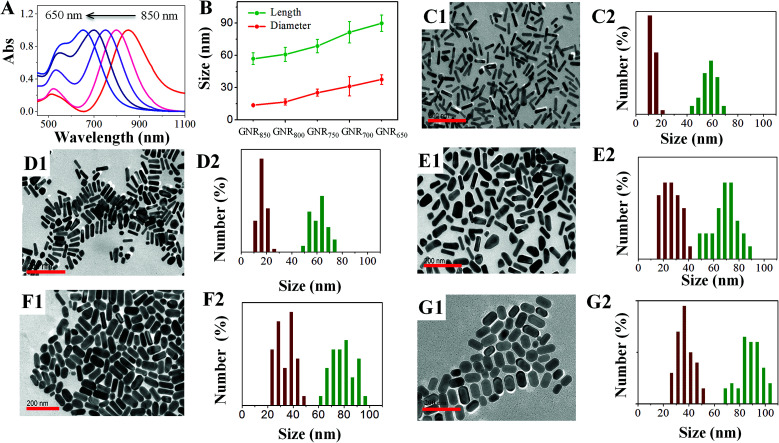
The normalized UV-Vis spectra (A), length and diameter (B), TEM images (1) and the corresponding size distribution (2) of GNRs with LLSPR at 850 nm (C), 800 nm (D), 750 nm (E), 700 nm (F), and 650 nm (G). The scale bar on each TEM image is 200 nm.

To validate our method, GNRs with designed LLSPR were obtained by controlling the use of HCl and sulfide. Through regulation, GNRs with LLSPR of 850 nm, 800 nm, 750 nm, 700 nm, and 650 nm were successfully prepared. The normalized UV-Vis absorption spectra and TEM images of these GNRs shown in Fig. 4 demonstrate that the LLSPR of GNRs can be continually, arbitrarily, and exquisitely tuned to any desired wavelength. In addition, the statistical lengths and diameters of these GNRs are displayed in Fig. 4B. These statistical results on lengths and diameters suggest the accurate control of GNRs with designed LLSPR and a small deviation (less than 3 nm), which is more precise than that obtained in some reported methods.^26,36^ These GNRs with high yields (∼95%) have well-defined morphology without the co-existence of bread-like and multipod-like nanoparticles. The TEM results showed that after adjusting LLSPR from 850 nm to 650 nm, the length of GNRs increased from 56.9 ± 5.5 nm to 89.9 ± 7.6 nm, while the diameter of GNRs increased from 13.6 ± 0.7 nm to 37.5 ± 4.5 nm. The growth along the longitudinal axis of GNRs (33.0 nm) was faster than that along the transverse axis (13.9 nm). The probable reason is the micelle structure of CTAB on the side of GNRs, which inhibits the growth along the transverse axis.^31^ After overgrowth, the aspect ratio reduced from 4.2 to 2.3, leading to a decrease in LLSPR. Therefore, our method for fine-tuning the LLSPR of GNRs is highly efficient.

### 3.3 Single GNR imaging

**Fig. 5:**
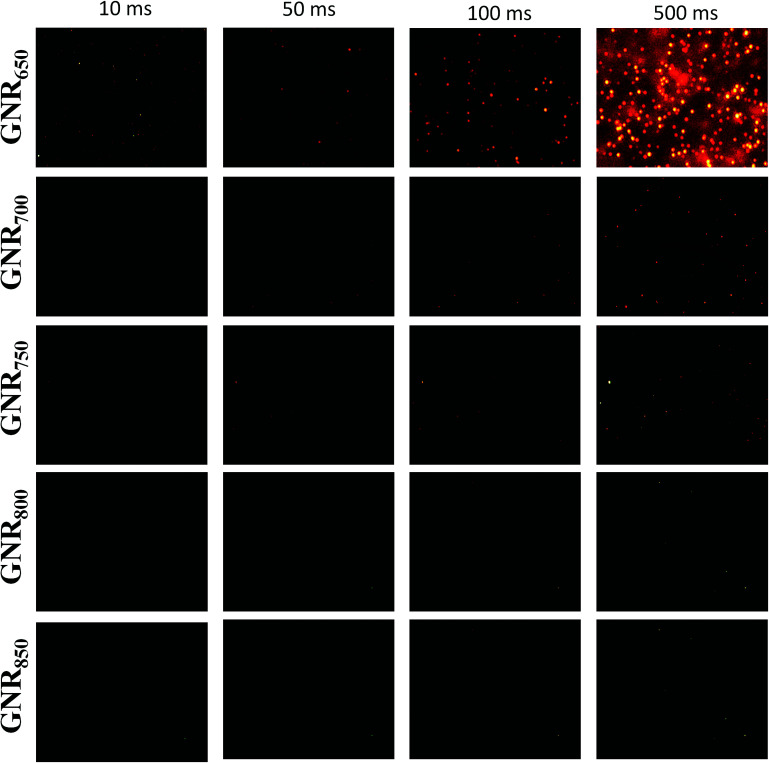
The imaging of single GNRs with LLSPR in the range of 650–850 nm under darkfield microscopy with an exposure time of 10 ms, 50 ms, 100 ms, and 500 ms, respectively. The sizes of each image are 16 × 12 μm.

Single nanoparticle imaging has become an important tool to investigate the dynamics of physical, chemical, and biological processes.^13,37^ The development of appropriate optical probes has been an important goal of nanomaterial engineering. Coincidentally, GNRs having the characteristics of tunable LLSPR and larger light cross-sections are excellent candidates.^1,5^ The darkfield images of GNRs with different LLSPRs were taken under different exposure times from 10 ms to 500 ms, as shown in Fig. 5. We observed only a small amount of GNR_650_ with an exposure time of 10 ms, and the brightness and number increased on increasing the exposure time. GNRs exhibit red colour because their LLSPR is in the range of red light (620–760 nm).^38^ The intensities of GNRs were different, further proving the unequal dimensions of GNRs. In contrast, GNR_700_ can be observed with the exposure times of 100 ms and 500 ms. However, GNR_750_ was only seen with a longer exposure time of 500 ms. For GNR_800_ and GNR_850_, no recognizable spot was observed even with 500 ms exposure time. GNR_650_ was 9.92 times brighter than GNR_700_ according to the images obtained for 100 ms exposure time, while this value was calculated to be 6.55 according to Gans' theory.^39^ It should be noted that the scattering intensity of a single GNR is related to both the scattering cross-section of GNRs and the overlap between LLSPR and light response of CCD. The scattering cross-section is generally proportional to the size of GNRs. Thus, GNR_650_ has a higher scattering cross-section than GNR_700_. On the other hand, the used DP73 CCD only has optical responses in the range from 400 to 650 nm. In comparison with GNR_700_, GNR_650_ fits the optical response of CCD better. Taken together, GNR_650_ is brighter than GNR_700_ and other GNRs.

To further confirm the importance of optical matching, we also prepared GNRs with LLSPR larger than 1100 nm, a diameter of 20 nm and a length of 358 nm (the average volume is 1.30 × 10^3^ nm^3^, which is larger than that of GNR_650_ with an average value of 9.93 × 10^4^ nm^3^) according to a previously reported method.^40^ As expected, these GNRs with large LLSPR were not observed even with an exposure time of 500 ms, further suggesting the significant role of the overlap between LLSPR and CCD response. As a result, GNR_650_ with high brightness obtained by our methods might be good optical probes for imaging, which can improve the localization accuracy and help monitor biochemical and physical events at a high speed. Moreover, our method is extremely suitable for engineering GNR imaging probes because it can not only adjust the LLSPR of GNRs to fit the optical response of detectors but also increase nanoparticle size associated with absorption and scattering cross-section.

## 4. Conclusions

In conclusion, we have reported a simple and reliable route to precisely tune the LLSPR of GNRs by HCl and Na_2_S-regulated overgrowth processes. In this assay, HCl decreased the overgrowth reaction rate by affecting chemical equilibrium, while Na_2_S stopped the overgrowth through the formation of Au_2_S to deplete the gold element. After a systematic study, 8.16 mM HCl was found to be optimal for suppressing the shift rate of LLSPR, and 1.83 mM Na_2_S was sufficient to stop GNR overgrowth efficiently. With this approach, GNRs with LLSPR at 850, 800, 750, 700, and 650 nm have been successfully prepared with an ultrasmall deviation of 3 nm. In comparison with other GNRs, GNR_650_ showed the strongest scattering under darkfield microscopy; it was 9.92 times brighter than GNR_700_ and the value was higher than the theoretical calculation value (6.65-fold). This is due to the satisfactory matching between the LLSPR of GNR_650_ and the response wavelength range of CCD. Through this post-synthesis method, however, only the preparation of blue-shifted GNRs compared to GNR seeds is possible. To obtain GNRs with longer LLSPR, GNR seeds with larger LLSPR need to be prepared first. Although it has some limitations, this method is highly valuable for the preparation of GNRs with tunable LLSPR and strong scattering, which should enhance their practical applications, *e.g.*, imaging and phototherapy.

## Conflicts of interest

There are no conflicts to declare.

## Supplementary Material
